# Supplementary material for: Rapid turnover of life-cycle-related genes in the brown algae
Source: Genome Biol. 2019 Feb 14;20:35. doi: 10.1186/s13059-019-1630-6 (PMC6374913; doi:10.1186/s13059-019-1630-6)
Supplement: Supplementary file 3 — Protein alignments, corresponding Gblocks html file and CDS sequences for the lineage conserved gametophyte-biased genes. (ZIP 2109 kb) [file 13059_2019_1630_MOESM3_ESM.zip › Con_GA_alignments/Ec-06_000170.aa_ali.fasta-gb.htm]

Ec-06\_000170.aa.fasta.trans.aa\_ali.fasta


## Gblocks 0.91b Results

Processed file: **Ec-06\_000170.aa.fasta.trans.aa\_ali.fasta**  
Number of sequences: **2**  
Alignment assumed to be: **Protein**  
New number of positions: **390** (selected positions are underlined in blue)

```
                         10        20        30        40        50        60
                 =========+=========+=========+=========+=========+=========+
Ec-06_000170     MAEPRQVTAKARADRTYDTPFTRETEPWGARTLVSVQPHHPDIPAVDHRSDVKFRRKQTL
MSTRG.33128.1    ------------------------------------------------------------
                                                                             


                         70        80        90       100       110       120
                 =========+=========+=========+=========+=========+=========+
Ec-06_000170     SVVSYNVLADSNSVRVRNCAPAVTSWGRRREVLLKEIFSVRADVLCLQDVDCFHQWWSPQ
MSTRG.33128.1    ------------------------------------------------------------
                                                                             


                        130       140       150       160       170       180
                 =========+=========+=========+=========+=========+=========+
Ec-06_000170     LTSAGYDSLFKQRTSRAAMHREGVVIAWKRDVFDLFRSGEMELNRLGEHEEDRSLAGKAA
MSTRG.33128.1    ------------------------------------------------------------
                                                                             


                        190       200       210       220       230       240
                 =========+=========+=========+=========+=========+=========+
Ec-06_000170     TSDNVALMTLLRPWQDSDHPSGACIVCTQLSEEEGYIGDAIRGLQAHGLTRSVEAFNSDF
MSTRG.33128.1    ------------------------------------------------------------
                                                                             


                        250       260       270       280       290       300
                 =========+=========+=========+=========+=========+=========+
Ec-06_000170     SLPIVMCGTMNCAPSSGTYEILCRGIEAQDPARPGPPGKPLVEPISTSAARVRWPAPSDD
MSTRG.33128.1    ------------------------------------------------------------
                                                                             


                        310       320       330       340       350       360
                 =========+=========+=========+=========+=========+=========+
Ec-06_000170     SESLSPAIDTYRLLWVPGGSRFLPGESVDAHEAGCVVYDLVKTESGRVRSEQNPLRAFVV
MSTRG.33128.1    --------------------------SVDAHEADCVVYDLVETESGNVRSEQNPLRSFTV
                                           ##################################


                        370       380       390       400       410       420
                 =========+=========+=========+=========+=========+=========+
Ec-06_000170     SGLSSGVAYEFRVSAINSLGQGPWSERSEPVRMPQMAGNDPVDKVLLGAASIKLLRKREL
MSTRG.33128.1    TGLSSGVAYEFRVSAINSLGQGPWSERSDPVRMPYIAGNDPEDKVLLGAASIKLLRKREL
                 ############################################################


                        430       440       450       460       470       480
                 =========+=========+=========+=========+=========+=========+
Ec-06_000170     EDARRRAEERLADPRAYELRKLVKVEGLLDLGMEKIHPFGSVSGVTPRYTDAALHPDLAN
MSTRG.33128.1    ENTRRRNEERLANPRAYEVQKLIKIDGLVDLDREDLHPFDSVSGLTPRYSDAMLHPDSAN
                 ############################################################


                        490       500       510       520       530       540
                 =========+=========+=========+=========+=========+=========+
Ec-06_000170     VRTDTGFPVIKAIEERRLSSGATGTTTRRRRQRKAKKTPRT--GSHQTRSDDEQVTSSST
MSTRG.33128.1    VRTDTGFPTLKATGESPLLP-VTKSGVRRQILRKSRGDANNSRACHGTRFDGDRHTSNSS
                 ##################    ############           ############## 


                        550       560       570       580       590       600
                 =========+=========+=========+=========+=========+=========+
Ec-06_000170     KG-LLDDDLSSLEDASAS---TTGGFRN-----------GSGEGRSDTSGVDTGESVTRP
MSTRG.33128.1    EAKHPQDGSSSLRHAQAWVLREAGGCAKNIEDRWHETKQGDRSVETDVHGSSDGESCCAS
                       ###########                      #####################


                        610       620       630       640       650       660
                 =========+=========+=========+=========+=========+=========+
Ec-06_000170     T-KSLLPP-------------KACGSSQREENILLRLDSLNVSGARSERQKHALGLRSAY
MSTRG.33128.1    TNLTVLPADGQAHAAGDVPTTSSSNSSEKDRSVLLRLEALNVSGARSERQKHALGLKSAY
                 #                        ###################################


                        670       680       690       700       710       720
                 =========+=========+=========+=========+=========+=========+
Ec-06_000170     MTHSSGGEPAFTMLSDTMVGTVDYIFFSADCLLPTQVLSIPEMGSLVRRDIQQTELSPAP
MSTRG.33128.1    MSYTSGGEPAYTTLSDTFTGTVDYIFFSADCLLPLEVLSIPEIGSLQGRDMQQTELVPTP
                 ############################################################


                        730       740       750       760       770       780
                 =========+=========+=========+=========+=========+=========+
Ec-06_000170     GLWAPSEWQGECGEERYMGEWSPYLRENPLRVKHRIPNELFPSDHLMLMANLVYFEPRCS
MSTRG.33128.1    GDLAPSEWRGTCDQDGYMGEWPSYLRENPLRVTRRIPNELFPSDHLMLMANLVYFEPRCA
                 ############################################################


                 
                 ====
Ec-06_000170     STWR
MSTRG.33128.1    STWR
                 ####
```

```
Parameters used
Minimum Number Of Sequences For A Conserved Position: 2
Minimum Number Of Sequences For A Flanking Position: 2
Maximum Number Of Contiguous Nonconserved Positions: 8
Minimum Length Of A Block: 10
Allowed Gap Positions: None
Use Similarity Matrices: Yes
```

```
Flank positions of the 6 selected block(s)
Flanks: [327  498]  [503  514]  [526  539]  [547  557]  [580  601]  [626  784]  

New number of positions in Ec-06_000170.aa.fasta.trans.aa_ali.fasta-gb:  390  (49% of the original 784 positions)
```
